# Sign2Story: A Multimodal Framework for Near-Real-Time Hand Gestures via Smartphone Sensors to AI-Generated Audio-Comics

**DOI:** 10.3390/s26020596

**Published:** 2026-01-15

**Authors:** Gul Faraz, Lei Jing, Xiang Li

**Affiliations:** Graduate School of Computer Science and Engineering, The University of Aizu, Aizuwakamatsu 965-8580, Japan; r1336004@u-aizu.ac.jp (G.F.); leijing@u-aizu.ac.jp (L.J.)

**Keywords:** Internet of Things, body area networks, gyroscope sensors, large language models (LLMs), generative AI, comics, voice, multimodal, accessibility

## Abstract

This study presents a multimodal framework that uses smartphone motion sensors and generative AI to create audio comics from live news headlines. The system operates without direct touch or voice input, instead responding to simple hand-wave gestures. The system demonstrates potential as an alternative input method, which may benefit users who find traditional touch or voice interaction challenging. In the experiments, we investigated the generation of comics on based on the latest tech-related news headlines using Really Simple Syndication (RSS) on a simple hand wave gesture. The proposed framework demonstrates extensibility beyond comic generation, as various other tasks utilizing large language models and multimodal AI could be integrated by mapping them to different hand gestures. Our experiments with open-source models like LLaMA, LLaVA, Gemma, and Qwen revealed that LLaVA delivers superior results in generating panel-aligned stories compared to Qwen3-VL, both in terms of inference speed and output quality, relative to the source image. These large language models (LLMs) collectively contribute imaginative and conversational narrative elements that enhance diversity in storytelling within the comic format. Additionally, we implement an AI-in-the-loop mechanism to iteratively improve output quality without human intervention. Finally, AI-generated audio narration is incorporated into the comics to create an immersive, multimodal reading experience.

## 1. Introduction

The recent advancements in generative AI for natural language processing have enabled large language models to understand human language commands or prompts and respond in a human-like manner. Various language models now exist to process text, speech, images, and even facial expressions. However, these models are still limited in their ability to understand non-verbal inputs that are used to convey messages without speaking or writing, such as hand movements or gestures. To address this limitation, we present a pipeline that combines multiple generative AI components, including a large language model, a vision–language model, a diffusion-based image generation model, and a text-to-speech system. This pipeline allows users to interact with AI through hand movements detected using onboard sensors, rather than traditional text or voice commands. Each component in the pipeline performs a specific role, collectively enabling end-to-end multimodal content generation.

The core motivation behind this system is to improve the accessibility of state-of-the-art AI technologies for users who may find conventional interaction methods challenging. Many existing systems require textual or voice-based commands, which may not be suitable for all users. By enabling gesture-based interaction, the proposed system aims to reduce barriers in AI-driven visual content generation and provide a more intuitive form of interaction.

In addition, this work serves as a test study for exploring how sensors embedded in common consumer devices, such as smartphones or smartwatches, could be utilized to support multimodal AI systems. The pipeline demonstrates how different components, including large language models, vision models, generative image models, and text-to-speech tools, can be integrated to perform a shared task. By relying on consumer-grade hardware and open-source AI models, the system remains flexible, extensible, and relatively easy to deploy.

To further improve output quality without additional human involvement, we incorporate an AI-in-the-loop mechanism. Specifically, a vision model (LLaVA:latest) analyzes the first generated manga panel and proposes prompt refinements for panels 2, 3, and 4. This design addresses a common limitation of diffusion-based image generation models: while they can produce high-quality images, each image is generated independently, resulting in inconsistencies across sequential panels. Although fixed random seeds can partially improve consistency, they are often insufficient when each panel requires a different prompt as part of a narrative sequence. By dynamically refining subsequent prompts based on the visual content of the first panel, the proposed mechanism improves visual coherence across the generated comic.

In summary, the key contributions and unique features of this work are as follows:

- The proposed system automatically transforms real-time news headlines into four-panel manga-style comics with consistent visuals, captions, and voice narration. This enables users to experience current events as story-driven content. Unlike prior approaches [1] that rely on pre-existing comics and manual annotations, our pipeline operates in a fully end-to-end manner, from news input to spoken comic output.
- Instead of relying on specialized or expensive hardware setups, such as VR headsets or external input devices (e.g., in [2]; for example, Meta Quest 3 and custom 3-key input), the system uses only a smartphone’s built-in gyroscope to detect hand movements. This approach is simple, affordable, does not require a camera, and can be deployed using widely available consumer devices.
- The pipeline supports customizable content selection through hand-based gestures and combines visual comics with synthesized speech to create an easily consumable audiovisual experience. This design could be explored as an alternative for users who may prefer gesture-based interaction over traditional methods.
- The system enables hands-free and non-visual interaction with real-time news content, allowing users to trigger, consume, and experience multimodal information without traditional input devices. This capability is not achievable by existing comic-generation or news-summarization systems in isolation.

The remainder of this paper is organized as follows. Section 2 reviews related work on sensor-based interaction, vision-based generative models, and their limitations. Section 3 describes the proposed methodology and details of the overall pipeline. Section 4 presents experimental results and example outputs, along with potential directions for future research. Section 5 concludes the paper and discusses key observations.

## 2. Related Work

In recent years, large language models have become powerful tools for understanding and generating text. With the advances in multimodal AI, these models can now work together with image and audio systems to create content across different formats. Researchers are now combining LLMs with diffusion models and vision models to produce creative results, such as turning written prompts into images, videos, or short stories.

Many studies have explored how AI can express storytelling elements through text or visual images.

For example, using prompt engineering, some models can generate pictures that match the mood or tone of a sentence. These systems show that AI can do more than just understanding and generating text; for example, they can also understand the mental state of the user.

The following studies explore related ideas and serve as comparisons with our work. They highlighted different approaches to integrating language understanding, visual generation, sign input, or contextual modeling. By presenting an overview of these works, we show how our proposed pipeline extends these methods by combining sensor input, LLM-based reasoning, and multimodal media generation in a single framework.

### 2.1. Mobile Sensor

While numerous previous studies have used mobile phones’ gyroscopes for PowerPoint remote controlled presentation, game-like health tracking systems, or device identification [3,4,5], all of them have represented significant advancements in entirely utilizing the device sensors and connecting to a remote server. However, most of them need the mobile phone and server to be connected over the same network, which can become a limitation for their use in real-world, distributed scenarios. Our approach leverages sensor input as an autonomous trigger for a multi-stage generative AI pipeline for content retrieval, image synthesis, visual reasoning, and speech generation, and performs all this via HTTP, so it can be accessed across different networks and locations.

### 2.2. AI Story and Image Pipeline

Recent work [6,7,8] has explored multimodal story generation using large language models, diffusion models, and even cooperative AI pipelines for story and image creation. However, these systems rely on static prompts and lack physical interaction, post-generation narrative refinement, and text-to-speech generation. Closer to our focus, MangaLMM [8] demonstrates the feasibility of manga understanding via VQA, but focuses on comprehension rather than generative storytelling; in contrast, our system uniquely combines sensor driven initiation, cross-network operation, manga-style synthesis, vision-guided narrative augmentation, and text-to-speech into a single end-to-end pipeline.

Gihyun Kwon and Jong Chul Ye [9] proposed a similar pipeline which focuses on generating a coherent visual storybook from a complete, pre-existing narrative. Their system used an LLM for prompt creation and uses an advanced diffusion model with “Iterative Coherent Identity Injection” to maintain a consistent character appearance across all images in the entire storybook. This is highly effective for adapting pre-existing stories. However, our study takes a different path by focusing on real-time, user-initiated creation rather than story adaptation. Instead of visualizing a story, our framework uses a physical gesture using a gyroscope sensor from a cellphone device and live news to generate a new, random narrative from scratch, which is then visualized as a multi-panel manga comic complete with AI-generated captions and audio. This shifts the focus from ensuring perfect visual consistency for a known character to creating a rich, multi-sensory experience from a simple user interaction.

### 2.3. Image to Story

Recent advances in multimodal AI models have made visual storytelling much better. Earlier systems often struggled to keep stories consistent or connected to the images. However, now, models like VIST-GPT [10] are trained to create narratives that stick closely to what is actually shown in the images. It uses special evaluation methods (RoViST and GROOViST) that focus on grounding, flow, and avoiding repetition, without needing human-written reference text.

Other models, like OMG-LLaVA [11] and VITRON [12], go even further, not only understanding entire images, but also specific objects and pixels within them. They can answer detailed questions, like “What is this character saying?” or “Why did they react that way?”, by combining vision and language at a very fine level.

### 2.4. Limitations

While recent advances in AI have unlocked impressive capabilities in text and image generation, it is important to recognize that these systems still have some limitations. One problem in diffusion models is their performance in numerical reasoning, which means that they cannot count or understand the numbers. A recent study [13] highlights this issue—when they gave prompts like “show 7 apples on the table” or “3 oranges and 4 bananas”, even the latest image generation models often failed to produce the correct number of objects. The problem becomes even more significant with increased complexity, and also, powerful models struggle to maintain accuracy. To address this, the authors introduced T2ICountBench, a new benchmark specifically designed to evaluate counting ability in text-to-image or diffusion models. Their results show that most models do not perform very well, suggesting that diffusion models are not yet reliable for tasks involving numbers.

This finding was a major factor in our design decisions. Initially, we considered generating a complete four-panel manga-style comic in a single image using a diffusion model. However, we realized that this approach can lead to inconsistent or incorrect panel structures, such as missing panels, duplicated content, and eventually, the unexpected result quality.

To ensure reliability, we updated our strategy. Instead of generating one entire comic image, we now generate four separate images, each representing a single story panel. These are later merged programmatically into a unified manga-style layout. This modular approach improves the quality and avoids counting while also giving us full control over the panel order, and ensures that each scene is clearly defined and visually distinct.

### 2.5. AI-in-the-Loop

Several works has explored iterative refinement loops where AI systems evaluate and improve their own outputs. For example, SELF-REFINE [14] shows that large language models can generate an initial response, critique it themselves, and then rewrite it, all without any human input or extra training. This idea of self-feedback matches our approach. Instead of text, we generate an image, then use a vision model to check if it matches the prompt, and update the prompt if needed. Similarly, generator evaluator frameworks, like dialog systems [15] and question generation [16], separate the creation step from the evaluation step, using the evaluator to choose or improve the output. These works are very similar when it comes to the AI to serve as an evaluator that proves automatic and AI driven feedback can enforce consistency and quality in generative systems, even without human input. Furthermore, one study [17] discussed the differences between human and AI-in-the-loop methods. In our case, we are not using a human to fix things, but in fact another AI (a vision model) as a helper. That makes our system an AI-in-the-loop setup, where the main AI (LLM generating prompts) is still in charge, but receives smart feedback from the other AI to stay on track.

As shown in Table 1, our proposed system differs from existing approaches of sensor inputs to story generation in terms of interaction modality, adaptability, and output generation.

## 3. Methodology

To implement the proposed approach in the experimental phase, smartphone sensors are utilized to capture and interpret hand movements using a mobile application that tracks sensor data and transmits it to the server. Modern smartphones are equipped with a variety of built-in sensors that provide information related to motion, orientation, and position, and are commonly used in applications such as GPS navigation, health monitoring, and proximity-based interactions [18]. For the purposes of this study, the smartphone device (Xiaomi Corporation, Beijing, China) is used specifically for its motion-related sensors. The choice of sensor for gesture recognition may vary across devices, and commonly available options include accelerometers, gyroscopes, rotation vector sensors, and gravity sensors.

The input gesture used in the experiments is a simple horizontal wave gesture characterized by a yaw motion around the device’s z-axis. This gesture was selected for its simplicity and reliability in triggering system actions. Although additional gestures can be implemented to support more interactions, the current implementation focuses on a single gesture to ensure robustness and low latency during evaluation.

**Figure 1:**
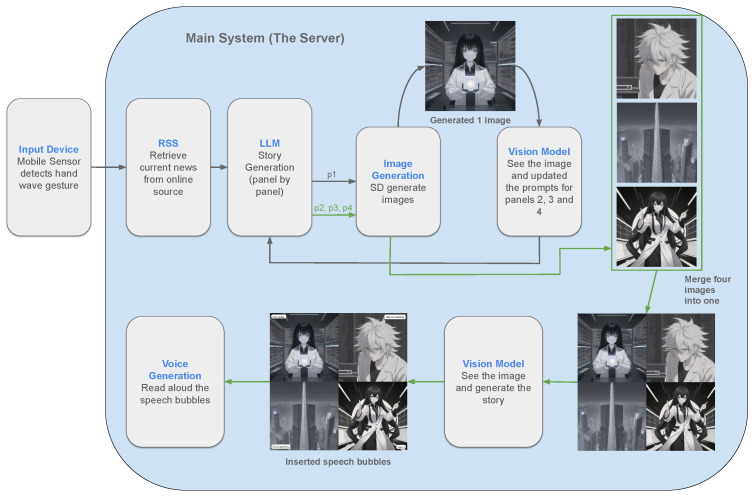
Illustration of full system pipeline.

Once the user input in the form of sensor signals reaches the server, it is processed by multiple models responsible for executing the specified content generation tasks. This procedure integrates gesture-based input with generative AI components to enable automated content generation. Figure 1 illustrates the overall system pipeline and data flow.

### 3.1. Mobile Application

For the mobile application, we incorporated Unity (version 2022.3.40f1 LTS, Unity Technologies, San Francisco, CA, USA) as the engine for developing an application in C# language, using the smartphone device as a sensor to detect hand movements. The app interface consists of a connection field for the server URL and a status indicator. Once connected, the app listens for gestures.

For a secure external access to our local server, we utilized the open-source tunneling service LocalXpose [19]. LocalXpose establishes an HTTPS tunnel from a local machine to the public internet, enabling remote clients to connect to a locally hosted endpoint without requiring complex network configuration such as port forwarding or DNS setup. This approach ensured encrypted communication, the stable exposure of our service during experiments, and accessibility for any device using the public URL provided.

**Figure 2:**
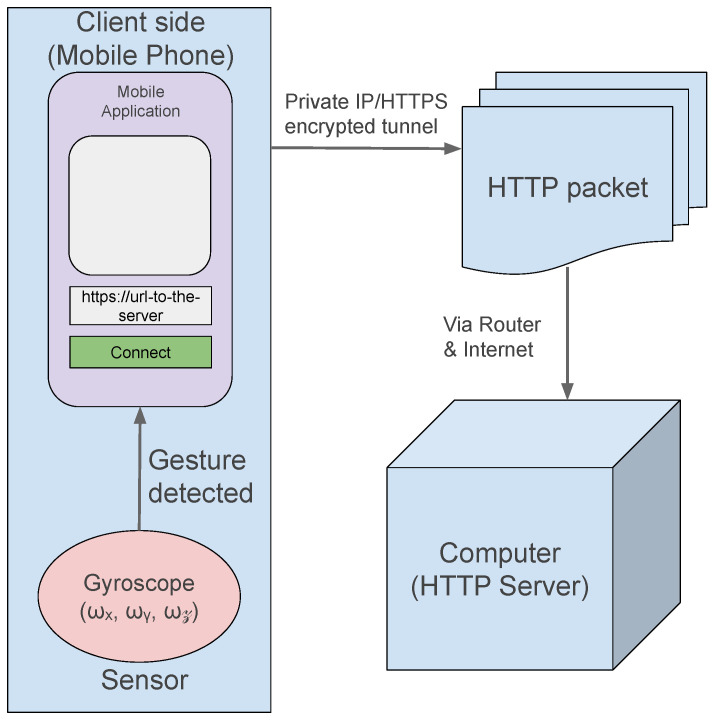
The client side, gyro sensor usage, and tunnel towards the server.

Figure 2 below shows an overview of the entire process:

### 3.2. Gesture Recognition and Sensing Method

Gesture recognition is implemented on a smartphone using the built-in gyroscope sensor. Angular velocity measurements are obtained via the device gyroscope and processed within a Unity-based mobile application. Sensor readings are captured in the Unity Update loop, resulting in an effective sampling rate of approximately 60 Hz for the tested mobile device.

The proposed method focuses on detecting horizontal wave gestures using gyroscope yaw rotation. At each time step, the yaw component of the angular velocity is extracted, and the change in yaw (Δyaw) between consecutive frames is computed. This operation serves as a lightweight preprocessing step to emphasize intentional rotational motion while suppressing slow drift.

Gesture segmentation is performed using a temporal windowing strategy. A gesture is initiated when the absolute value of Δyaw exceeds a predefined threshold (τ=1.2rad/s). Alternating left and right rotations are tracked, and a wave gesture is confirmed when two directional swings are detected within a maximum duration of 1.5 s, with a minimum interval of 0.2 s between consecutive swings.

Gesture recognition is realized through a rule-based decision mechanism rather than a learned classifier. The system is not designed to recognize structured sign language, but instead detects simple motion-based gestures that are used to trigger predefined system actions. Once a wave gesture is detected, a predefined command is transmitted to the server to trigger the content generation pipeline. This design avoids on-device training overhead while ensuring low latency and reproducible gesture-based interaction.

The horizontal wave gesture was selected due to its simplicity, ease of execution, and robustness to variation across users. It requires minimal physical effort, does not depend on precise finger articulation, and can be reliably detected using a single gyroscope axis. These properties make it suitable for rapid and low-latency interaction in consumer mobile devices.

The proposed gesture recognition method assumes that the mobile device is held in hand and that the user performs the gesture with a moderate and deliberate motion. Sudden involuntary movements, device shaking, or changes in device orientation may affect the detection accuracy. In addition, the current implementation method supports a limited set of predefined gestures, prioritizing reliability and low latency over expressive gesture vocabulary.

### 3.3. Sensor Input Method

The mobile application acts as a bridge between the smartphone sensors and the server by capturing sensor input and transmitting it for further processing. In most modern smartphones, the gyroscope sensor is a standard component and is often paired with the accelerometer to enable motion-aware interactions. Traditionally, gyroscopes are used for screen orientation, image stabilization, and gaming. However, recent work has explored their potential for gesture-based user interfaces [20].

In the proposed system, the smartphone’s built-in gyroscope sensor is used to capture motion during user interaction. The gyroscope measures angular velocity around the three principal axes (x, y, and z), providing high-frequency data that reflects real-time changes in device orientation. This information allows the system to detect intentional motion patterns generated by the user.

### 3.4. Server Architecture

This movement signal is recognized by the application and it sends a message to the server. Once the message is received, the main process in initiated. It starts with using RSS to retrieve the latest tech news from Google. The reason behind using news was to obtain a unique response and a different story basis each time. This news is then provided to the large language model to generate a prompt for the diffusion model which includes a story for the comic; for example, Ref. [21] experimented with different prompt structures, altering the sequence of output instructions and including explanatory reasons. In our case, we used the prompt engineering to some level and presented the prompt as if the model acts as a storyteller and writes a story on the basis of the provided news.

Furthermore, we tried to generate a single prompt for the entire story and send this to the diffusion model to generate a 4-panel manga style comic; however, to improve the quality [13], we break down the prompt into 4 parts to ensure consistency and output generated from the diffusion model. The final version of the generated prompt is as follows:

- Panel 1: A weary police officer, slumped at their desk under harsh fluorescent lighting, stares blankly at a stack of documents, rendered in detailed cross-hatching and conveying exhaustion.
- Panel 2: A holographic projection of swirling data streams and translating symbols hovers above a futuristic console, sharply angled to emphasize its technological sophistication.
- Panel 3: The officer’s eyes widen with surprise, depicted with exaggerated manga-style anime eyes, as he examines a translated audio transcript on their screen.
- Panel 4: A determined officer strides purposefully through a bustling city street at dusk, lit by neon signs, showcasing a dynamic, three-quarter pose and bold outlines.

In this case, the news story was as follows:

- New A.I. translation technology helps police officers—NBC News

**Figure 3:**
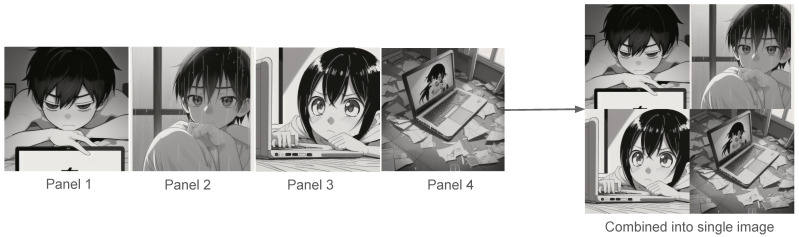
All 4 panels are created one by one and combined at the end.

This breakdown helped in obtaining better-quality results from the diffusion model, as these panels are sent to the model [9] one by one via API, and a set of 4 different images is created, which were later combined in a 2 × 2 layout in a programmatic way inside the server code. Figure 3 shows the results.

### 3.5. Problems and Solutions

While the initial image quality from the diffusion model was good, we noticed a major issue: the images were not consistent with each other, and it was noticed that they belong to other stories. Since the diffusion model generates each image independently, every new prompt produced an image that looked like it came from a completely different story. The characters, styles, and details did not match.

We tried using ControlNet and fixed seed values to maintain consistency, but without a reference image, the ControlNet method was not able to achieve the results we wanted. To solve this, we introduced another vision model into the pipeline. After the first image is generated, the vision model analyzes it and then adjusts the prompts for the second, third, and fourth images. This way, the first image becomes the reference image for the others, and the vision model acts like a supervisor, checking each new prompt and modifying it whenever something does not match the reference. With this approach, the remaining images stay visually aligned with the first one, greatly improving the consistency across the whole sequence.

Now, we have a 4-panel manga style comic ready, but it is currently without labels. It is just an image, so it is difficult to understand for a user. A vision model is utilized in the next part of our pipeline.

The role of the vision model is to view the image and generate a story around it. We use this model instead of relying on the one which created the image, because at this stage, we already have a visual reference as the image, and the story must stay consistent with the image. If we had simply used the story produced earlier (before the image was generated), the result might have aligned with the original news input, but not have matched the visual details in the final comic. Once the story is generated on the image, instead of displaying the entire story to the user simultaneously with the image—as this would downgrade the user experience, with the need to watch the image and read the story at once— we let the model generate the story so that we obtain the panel-wise captions. Now, these captions are inserted on each image using the program inside the server again in the form of speech bubbles, which gives the result a real feel of a manga-style comic. As we understand, the diffusion model has challenges that emerge in applying diffusion models to text generation, which include the discrete nature of textual data [22], making it hard to generate human-readable text while generating images.

Additionally, in Ref. [21], prompt optimization proved beneficial in aligning LLM outputs with ground-truth scores, which demonstrates the need for sufficient data samples for arbitrary scoring tasks. In our case, the instructions contain the message to the models, the size and number of total words and tokens, and the rules which specify exactly where to start and end. This depends on which diffusion model is in use, and the number of token can vary accordingly. For instance, the stable diffusion model SDXL accepts 75 tokens for one prompt, which means that if the limit is exceeded, the upcoming tokens will be ignored, and this will impact the result.

### 3.6. Text-to-Speech

The final step involves generating voices against the speech bubbles. For this purpose, we employed GPT-SoVITS (available online: [23]). As our goal is to build the entire pipeline using open-source and freely accessible tools, we trained this model for voice generation to ensure accessibility for users who may not afford paid APIs. The model was trained using the LJ Speech Dataset (available online: [24]), which is a public-domain speech dataset. After the entire process, both the generated image and the audio are presented to the user and locally stored on the server.

### 3.7. Output

**Figure 4:**
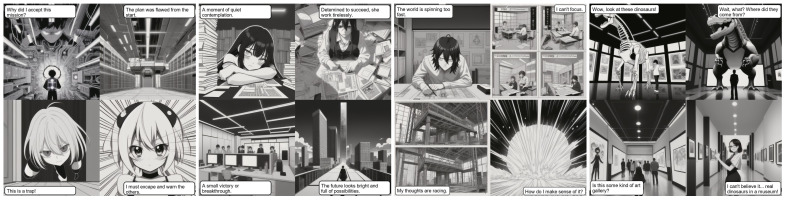
Illustration of results.

Figure 4 shows some of the results of experiments using the kawaiiRealisticManga v04 diffusion model.

## 4. Experiments and Results

In this section, we evaluate the performance of our proposed pipeline in automated comic generation. Our experiments are designed to test how well the system integrates sensor input, LLM-based reasoning, visual content generation, and speech narration. We start by describing the smartphone application, which collects real-time sensor data, then presents the results from the AI pipeline, including visual output quality and generation speed. This structure allows us to highlight both the individual components and the overall effectiveness of the complete system.

### 4.1. Smartphone Application

**Figure 5:**
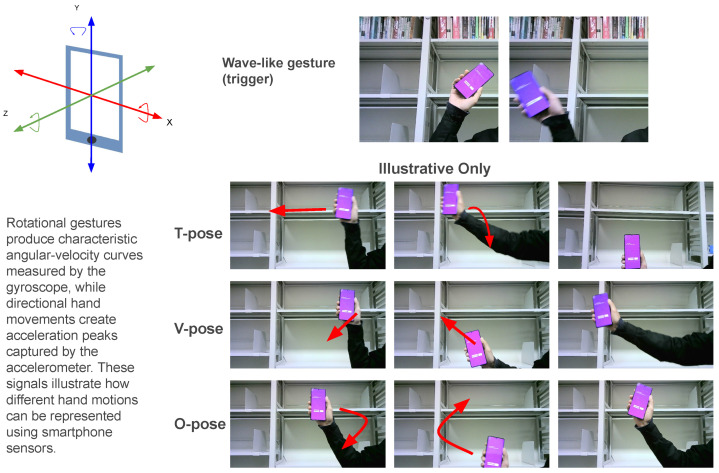
Illustrative examples of simple hand configurations and a wave-like motion used to demonstrate user-friendly gesture design. In the current implementation, only a wave-like gesture (similar to an ASL “Hello”) is used as the system trigger; other hand shapes (e.g., T, V, O) are shown for illustrative purposes only and are not recognized by the system.

Our experiments began with the smartphone application developed using Unity (2022.3.40f1) and Android 15. We implemented a C# script to collect the input from the device sensor. Specifically, we used the gyroscope to detect the short wave-like movement and rotations of the device, which correspond to hand motions in our pipeline. These sensor readings serve as the initial input for our system, enabling the LLM and visual modules to generate context-aware comic content. Figure 5 illustrates the hand wave gesture characterized by a yaw rotation, as captured by the smartphone gyroscope during user interaction. Although multiple hand shapes are illustrated to demonstrate gesture simplicity, the current system only implements and evaluates a single wave-like gesture as the trigger.

### 4.2. Gesture and Output Quality Evaluation

To quantify the reliability of our gyroscope-based wave detection algorithm, we conducted a controlled evaluation comprising 50 trials. These included 35 intentional wave gestures performed at varying speeds, and 15 non-gesture movements simulating natural phone handling (walking motions, device rotation, and random movements). The system correctly identified 28 of 35 wave gestures (true positives) and correctly rejected 12 of 15 non-gesture movements (true negatives), resulting in 3 false positives and 7 false negatives. The resulting performance metrics are summarized in Table 2.

To quantitatively assess the semantic alignment between the input news content and the generated comic narratives, we employ an LLM-based [25] relevance scoring approach. For each sample, an independent large language model (Deepseek-R1) evaluates how closely the generated story reflects the news story provided. The semantic relevance score is interpreted on a three-level scale, which is basically scored between 1 and 5. Scores of 4–5 indicate high relevance, where the generated story closely reflects the key events and semantics of the input news. A score of 3 indicates moderate relevance, where partial alignment is observed, and scores of 1–2 indicate low relevance, corresponding to weak or minimal semantic alignment. The results are summarized in Table 3.

Visual consistency across consecutive comic panels is evaluated using a CLIP-based [26] embedding similarity metric. Each panel is encoded using a vision–language model, and cosine similarity is computed between consecutive panels within a comic sequence. The final inter-panel visual consistency score (between 0 and 1) is obtained by averaging these similarities. The results are reported as mean and standard deviation across ten experiments, where higher scores indicate greater semantic and stylistic consistency across panels.

### 4.3. Server

The next and most critical component of the system is the server, which is developed using the Python 3.10 programming language. This server hosts all models and system components and coordinates their execution during the content generation process. For the experimental setup, a laboratory desktop computer running the Windows 10 Pro operating system was used as the server.

The server begins listening via port 9000. Through localXpose, this port becomes accessible externally with the provided public IP address. When the user performs the wave, the smartphone application confirms the action and sends a request to the provided exposed address. Once the server receives the request, it initiates the next stage of the pipeline. The first operation in this stage is retrieving the latest technology-related news from Google News RSS. From these headlines, we extract the top news items of the day. To generate a diffusion model prompt for image creation, the system concatenates this selected news headline with a predefined prompt template. This template has been optimized [21] in advance and includes detailed instructions that guide the large language model toward producing the desired output, which will act as a prompt for the diffusion model.

**Figure 6:**
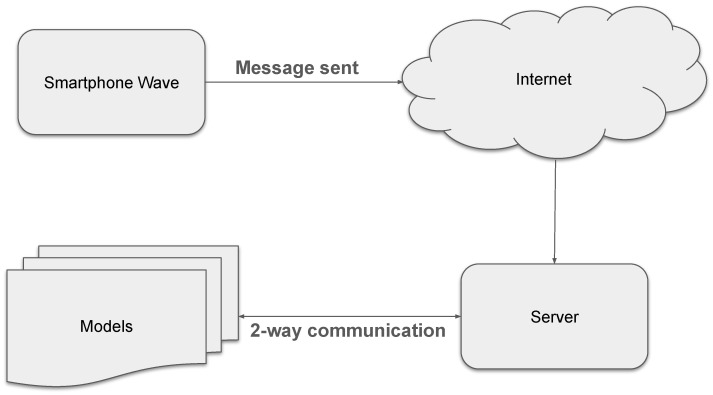
This visualizes the position of the server in the system communication with multiple components at a time.

For our experiments, we have incorporated some open-source models (LLaMA3.2, Gemma3, Qwen3) with 3b, 12b, and 32b parameters for this stage. Figure 6 illustrates the overall workflow of the system.

### 4.4. AI in Action

Since the major functionality in the experiments belongs to generative AI models, we have shortlisted some of the models on the basis of their balance between performance and computational efficiency, which makes our pipeline suitable for real-time application with limited hardware requirements and availability, as they are all open-source. The pipeline starts with an LLM, which is supposed to generate a prompt ready for the diffusion model which explains the story, and information like, structure, characters, scene, and background to generate quality images, because the better the prompt is, the better the results will be. The server sends an initial prompt template with the news headline attached containing instructions like the objective, context, and format specifications of the output.

The response generated by the language model is used as the prompt for the diffusion model in the system. The server acts as a central coordinator, automatically dividing the generated content into four panel descriptions, each containing scene-specific details for image generation without human intervention. The prompt for the first panel is directly sent to the diffusion model to generate the initial image, while the remaining panel, prompts are temporarily held for further refinement by the AI-in-the-loop process. Based on the provided prompt, the diffusion model generates an image using 35 sampling steps per panel.

Once the first image is generated, a vision model inspects the output and adjusts the prompts for Panels 2, 3, and 4 to ensure visual consistency across character appearance, style, and scene attributes before the remaining images are generated.

In the next step, these four generated images are merged into one manga-style image, and later, the text bubbles or labels are inserted with the help of an open-source library called Pillow, which has compatibility with Python 3.

Since the system created the image, before the display, it needs a story to make it look like a manga-style comic. For this purpose, we integrated a vision model to overview the image and plan a story. There is a predefined template that is optimized in a way that it can make the model see all the four panels, find a relation between the images, and generate a story in a fun way, and the generated image is attached to this template and sent to the vision model. The model outputs speech-bubble text for the comic panels; however, before insertion, the server segments the generated content into four parts, following the same panel-wise division that was applied earlier to the LLM-generated prompts. These four speech bubbles are then inserted into their corresponding panels in the final image.

Now, the system prepared the output, but along with showing the image with the speech bubbles, we performed another experiment which makes our system a fully fledged automated [27,28] version, and this step involves a process of text-to-speech conversion. There are multiple text-to-speech models and tools present in the market, and using a pre-trained model API was the most convenient option for this project because it automatically converts the text to speech and plays the audio once the image is displayed without any training. However, since our vision is to make the entire system based on open-source tools and models, we picked gpt-soVITS for this purpose and trained it using a dataset called LJ Speech Dataset, which is also freely available for research purposes. There is no need to further modify the text generated by the vision model [29], as it is already well structured based on the optimized prompting strategy [21]. The response is then segmented into four concise sentences, each corresponding to a panel, to prepare the input for the text-to-speech module. Finally, both the generated image and the synthesized audio are presented to the user.

### 4.5. Failure Cases

While the AI-in-the-loop mechanism significantly improves consistency, certain failure modes persist. We systematically analyze these failures and propose mitigation strategies.

**Figure 7:**
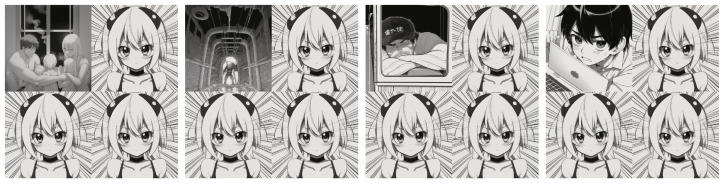
These images are generated from entirely different news stories at different times, but the character remains the exact same.

**Observation:** in approximately 20% of the generated results, the diffusion model produced identical characters across multiple panels (Figure 7), despite the differences in context.

**Root Cause Analysis:** this occurs due to the following factors:

**Training data bias:** the model was trained on datasets containing prominent recurring characters.

**Seed locking:** fixed random seeds combined with similar prompt structures can converge to identical outputs.

**Mitigation strategy:** we implemented prompt diversification along with random character attributes like hair style and colors into panel prompts after the first image generation. Additionally, we updated the value of random seeds for each panel generation while maintaining style consistency.

### 4.6. Latency and Runtime Stability Evaluation

To evaluate runtime stability, we conducted 10 repeated end-to-end experiments under identical hardware and model configurations. The proposed system achieved an average execution time of 53.98 s, with a minimum of 46.46 s and a maximum of 61.47 s. To quantify temporal consistency across runs, we computed the Root Mean Square Error (RMSE), obtaining a value of 5.02 s. This low RMSE indicates limited variance in execution time despite the multi-stage pipeline involving language generation, image synthesis, vision-based refinement, and audio narration, supporting the system’s near-real-time interactive performance with stable operational behavior.

(1) $$RMSE=\sqrt{\frac{1}{N}\sum_{i=1}^{N}{\left(T_{i}-\bar{T}\right)}^{2}}$$

where $T_{i}$ denotes the total end-to-end execution time of the *i*-th experiment, $\bar{T}$ represents the mean total execution time across all experiments, and *N* is the number of repeated experimental runs (N=10). The end-to-end runtime stability results across repeated experiments are summarized in Table 4.

End-to-end system latency is defined as the time elapsed from the moment that a gesture is detected on the mobile device to the moment the generated comic image is displayed and audio playback begins. This measurement includes sensor processing, server communication, content generation, and result delivery, but excludes background application startup time. Furthermore, all experiments and qualitative assessments were conducted by the authors, and no formal user study involving end-users or participants with disabilities was performed. The average latency at each stage of the end-to-end system pipeline is summarized in Table 5.

### 4.7. Results

To evaluate pipeline performance under different computational conditions, we conducted multiple experiments using various models and hardware configurations. The initial experiments generated the entire comic as a single image, which served as a baseline for comparison. Performance testing across different GPUs revealed significant variance in processing times: an RTX 3090 Graphics Processing Unit (GPU) (NVIDIA Corporation, Santa Clara, CA, USA) with 24 GB of Video Random Access Memory (VRAM) completed the end-to-end process in approximately 50 s on average, while an RTX 4090 (24 GB VRAM) (NVIDIA Corporation, Santa Clara, CA, USA) achieved this in 24.72 s. Each configuration was tested 11 times to ensure measurement reliability.

The following sections detail specific experimental scenarios and their outcomes:

#### 4.7.1. Experiment Scene 1

**Hardware Properties and Execution Time:** the process utilized a NVIDIA GeForce RTX 3090 (24 GB VRAM) and took 1 min and 01 s to finish the process.

**News Headline:** a statement by the CEO of Google on how vibe coding can help non-tech graduates to build careers in technology was crawled as input.

**LLM Output:**

- Panel 1: generate a single black-and-white panel manga-style comic depicting a young graduate, eyes wide with excitement, leaning forward intensely in a brightly lit, modern tech office, and a complex code interface displayed on a large screen behind him, utilizing dramatic shading and a dynamic pose to emphasize their focus.
- Panel 2: generate a single black-and-white panel manga-style comic showing a CEO calmly gesturing with both hands, bathed in soft office lighting, surrounded by a diverse group of young professionals, displaying a wide shot emphasizing the collaborative atmosphere.
- Panel 3: generate a single black-and-white panel manga-style comic showcasing the graduate meticulously typing on a keyboard with speed lines illustrating rapid coding, set in a slightly low-angle shot to highlight their determined effort and skill.
- Panel 4: generate a single black-and-white panel manga-style comic showing the officer giving a slight nod of approval, with a dramatic spotlight shining on him, and a close-up of their face conveying trust and optimism, emphasizing a powerful, confident expression.

Using these panels, the system generated the first image, and the LLaVA:latest vision model supervised this image. To maintain consistency, it rewrites the upcoming panels 2, 3, and 4 as

**Rewritten Prompts:**

- Panel 2: create a black-and-white panel manga-style comic of a CEO calmly gesturing with both hands while standing in front of a diverse group of young professionals, all looking up at them in an indoor office environment during the day. The lighting is diffused across the room, emphasizing the collaborative atmosphere. Maintain the close-up shot of the officer’s face and upper body, and make sure that their facial expression reflects optimism and trust.
- Panel 3: create a black-and-white panel manga-style comic of a young person sitting at a desk, meticulously typing on a keyboard with speed lines illustrating rapid coding. The person is dressed in casual attire and is bathed in focused light from above, highlighting their determination. The environment suggests an indoor setting, possibly a living room or a cosy corner of an office.
- Panel 4: create a black-and-white panel manga-style comic with the officer smiling warmly at the camera from an elevated angle, emphasizing trust and optimism. The focus should be on him, capturing their face and upper body. The background is not important, but can suggest an indoor setting during the daytime with soft, natural lighting.

This result captured the technical elements of the original news story in a visually structured format.

**Vision Model Output:** the model generated a different, story looking set of dialog scenes:

- “I cannot believe they’re doing this to me!”
- “This is going to be a long day.”
- “Maybe if I just sit here and keep quiet…”
- “Looks like I’ll have to use some strategy.”

**Figure 8:**
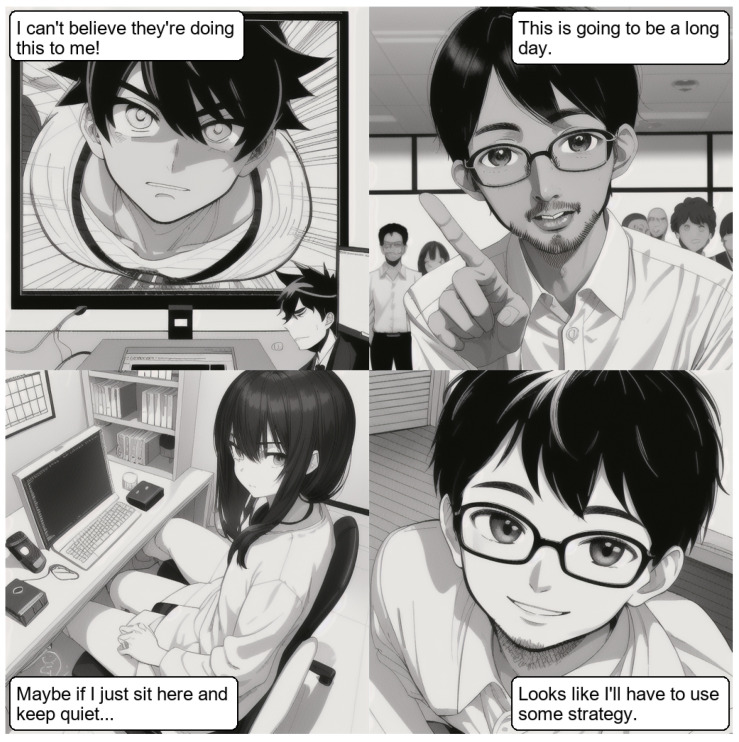
Output image generated by the pipeline.

Figure 8 shows the output of this experiment using Advanced Stack and a supervisor model.

#### 4.7.2. Experiment Scene 2

**Hardware Properties and Execution Time:** the process utilized a NVIDIA GeForce RTX 3090 (24 GB VRAM) and took 1 min and 12 s to finish the process.

**News Headline:** new technology a is ’game changer’ for stadium fans, say experts.

**LLM Output:** the model was prompted to generate a comic strip panel by panel. The model responded with the following detailed panel breakdown:

- Panel 1: generate a single black-and-white panel manga-style comic depicting a frustrated fan slumped in a stadium seat, illuminated by harsh overhead lighting, showcasing a dynamic, slumped pose with dramatic shading to emphasize disappointment, viewed from a wide shot.
- Panel 2: generate a single black-and-white panel manga-style comic showing a technician smiling intently while adjusting a futuristic headset in a brightly lit control room, using speed lines to convey technological progress, captured in a close-up with focused attention.
- Panel 3: generate a single black-and-white panel manga-style comic portraying a surprised fan looking up in amazement while wearing the headset, bathed in a soft glow, utilizing a dramatic, upward-angled perspective and thick outlines.
- Panel 4: generate a single black-and-white panel manga-style comic showing a jubilant fan cheering with arms raised amidst a vibrant, cheering crowd, captured from a sweeping, over-the-shoulder perspective, complete with energetic speed lines and high-contrast shading.

Using these panel prompts, the system generated the first image, and the vision model visualizes this image, rewriting the upcoming panels 2, 3, and 4 as

**Rewritten Prompts:**

- Panel 2: a black-and-white panel manga-style comic shows a technician sitting at a desk with their glasses perched on top of their head, adjusting a futuristic headset. The technician’s hair is tousled slightly from moving around, indicating focus and concentration. Speed lines radiate outward from the technician, suggesting rapid progress or an intense thought process. The room is softly lit from the left side, emphasizing the technician’s focused expression.
- Panel 3: a black-and-white panel manga-style comic captures the moment when the fan, wearing the headset, is hit by an unexpected sensation, as indicated by their wide eyes and upward gaze. The dramatic perspective suggests an intense emotional experience. A soft glow surrounds the fan, symbolizing surprise or amazement. The room’s ambient light casts a warm glow from the left side, highlighting the fan’s reaction.
- Panel 4: a black-and-white panel manga-style comic shows a jubilant fan, still wearing the headset, cheering with their arms raised amidst a vibrant crowd of other fans. The energetic speed lines and high-contrast shading convey the lively atmosphere of the event. The room’s perspective shifts to an over-the-shoulder angle, giving a sense of the fan’s excitement as they join in the cheering with the crowd.

**Vision Model Output:** the model generated a different, more playful set of dialog-driven scenes:

- “This is my dream, just like theirs.”
- “I can’t believe I made it to the big stage!”
- “They look so cool! Maybe I should ask for advice.”
- “It’s finally happening! My heart is racing with excitement!”

**Figure 9:**
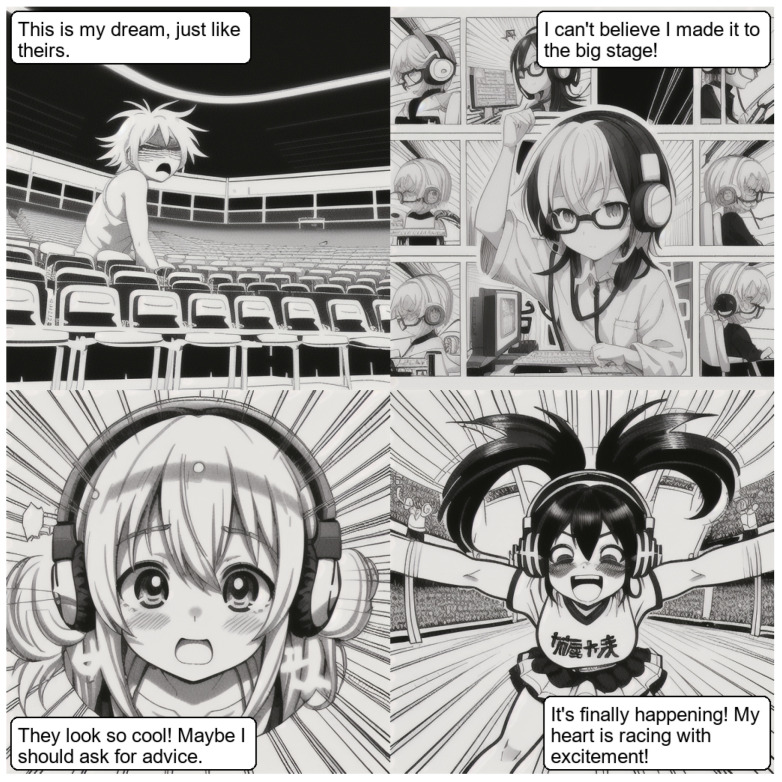
Output generated by the pipeline using moderate-sized models.

Figure 9 shows the output of this experiment using Advanced Stack and a supervisor model.

#### 4.7.3. Observations

- Although Qwen gives promising results, using the LLaVA vision model for both stories from image generation and supervision reduced the total execution time, and resulted in the quality staying consistent and moderate.
- Smaller model offered a dialog-based comic flowing away from the original topic and moving towards the other side of the image. This shows that the different number of parameters have a huge impact on the result.

### 4.8. Output Delivery and User Interface

The smartphone application serves primarily as a gesture-sensing input device. When a gesture is detected, the mobile app sends an HTTP request to the server, which then executes the multimodal generation pipeline. All AI processing occurs on the server side, with the generated comic and audio files saved locally on the server machine.

Upon completion of the pipeline, the system automatically performs the following:

- Displays the final comic by opening the generated image file using the system’s default image viewer.
- Plays the audio narration concurrently using the Windows sound API (winsound).

The user experience is thus a seamless transition from performing a gesture to receiving both visual and auditory output, with the comic appearing as a standalone image window and the audio playing through the system speakers.

This lightweight approach demonstrates the feasibility of sensor-driven content generation without complex client-side rendering or dedicated user interfaces, making the system easy to deploy and test across different environments.

### 4.9. Discussion

This project demonstrates the potential of combining large language models with physical sensors to understand the command from the user or prompt without directly interacting. In these experiments, we have incorporated multiple models (language and vision), and a text-to-speech model, with the initiating process using a physical sensor. From this, we can estimate that this can perform significantly more tasks using different combinations of hand gestures and LLMs using such multi-modal pipelines, which are feasible even on consumer-grade hardware. Furthermore, we used AI-in-the-loop to make the result quality better. Some of the pros and cons are discussed below:

### 4.10. Pros and Cons

- The integration of multiple models for text, vision, and image generation in the pipeline made the further customizations easier.
- Despite limited VRAM, the process still performed fine and did not crash, showing good resource compatibility.
- Although the pipeline operates successfully on limited hardware (8GB VRAM), the processing time of approximately 1 min and 42 s presents a latency challenge for scenarios requiring immediate feedback.
- Diffusion models sometimes misinterpret the prompt. This can be improved by further prompt tuning. For example, diffusion models struggle when it comes to counting or other numerical tasks [13] and hallucinate, which can damage the result quality or generate identical panels. However, this could be a possible research direction for further enhancements.

### 4.11. Comparison

We performed multiple experiments using different models; however, the final quality and consistency among the images in the results are mainly based on the vision model that modifies the prompts to match the images. We have utilized some models and compared the results in Table 6.

Image accuracy refers to the degree to which generated images visually reflect the entities and events described in the corresponding news headline or story. Consistency is evaluated based on the continuity of character appearance, visual style, and scene elements across all panels within the generated images. Qualitative comparisons in Table 6 and Table 7 are reported using a three-level ordinal scale (high, moderate, low). For image-related evaluations, ‘high’ indicates clear visual detail, the accurate depiction of described entities, and consistent character appearance across panels; ‘moderate’ indicates partial consistency or minor visual artifacts; and ‘low’ indicates frequent artifacts or inconsistencies. For story generation evaluations, ‘high’ indicates coherent narrative flow, representing an accurate reflection of the input news content, and sufficient descriptive detail; ‘moderate’ indicates partial coherence or reduced detail; and ‘low’ indicates fragmented narratives or semantic inaccuracies. All qualitative assessments were conducted by the authors through visual and textual inspection.

Given the exploratory nature of this work and its focus on system feasibility and end-to-end behavior, author-based qualitative inspection was considered sufficient to support the limited comparative claims made in this study.

We have also compared the models to overview the news story generation, and we show some comparisons in Table 7.

All qualitative evaluations are reported using a three-level ordinal scale (high, moderate, low). Any legacy labels from preliminary analyses were normalized to this scale for consistency.

### 4.12. Limitations and Discussions

The qualitative evaluations reported in Table 6 and Table 7 were conducted by the authors using a three-level ordinal scale. As with any author-based qualitative assessment, this introduces a potential risk of subjective bias. However, the purpose of this evaluation is exploratory, aiming to analyze relative trends and system behavior rather than to provide definitive or statistically generalizable comparisons. Accordingly, the qualitative results should be interpreted as indicative rather than conclusive.

While this study presents the foundational pipeline for the automated system that is using a combination of large language models and diffusion models along with hand gesture, it also opens several doors for future works that include enhancements and improvements. The basic part that can be enhanced is creating an architecture that can retain storage so it can keep the previous record and memory to keep the context for generating the better results. The other part includes the feedback mechanism, as the current system is unidirectional, meaning that incorporating the user feedback or adaptive refinement loops can make the system interactive, allowing real-time editing or re-generation based on user preferences that could significantly improve usability. In addition, minor changes, like using different language, vision, and diffusion models, can be used to explore a huge difference in quality of results. Furthermore, multiple hand gestures, including movements of fingers, can be explored by using different equipment, like hand-gesture-specific sensors and cameras. Future research could also investigate domain-specific fine-tuning and dialogs between the models that can go beyond our expectations, including quality and user engagement.

## 5. Conclusions

This research presents a functional, lightweight, and hardware-friendly multimodal content generation pipeline that leverages real-time hand motion data captured via embedded motion sensors in a smartphone as the primary input modality. The system integrates compact large language models, a diffusion model for high-quality image generation, and an open-source text-to-speech framework.

The experimental results highlight the importance of model selection within the pipeline, as different combinations of language and vision models affect performance and output characteristics. While LLaMA demonstrated strong contextual accuracy when processing visual inputs, LLaVA contributed a more expressive and conversational style, enhancing storytelling flexibility. Future work may explore tighter integration between language, vision, and diffusion models, as well as fine-tuning strategies to support additional customization, such as varying comic styles.

Furthermore, by relying on widely available smartphone sensors and network-based communication rather than specialized hardware, the proposed approach is motivated by the goal of lowering the barrier to interactive content generation and exploring multimodal storytelling experiences with everyday consumer devices.

## Figures and Tables

**Table 1:** The table shows the basic differences between our system and other story generation systems.

Feature	Prior Works	Our Work
Input Method	Text prompts or pre-written stories/images [6,7]	Uses mobile sensor input + live news
User Interaction	Static: one prompt –> output [6,9]	Dynamic and physical: user waves phone –> starts full AI pipeline
Story Generation	Based on fixed prompts or existing narratives [6,7,9]	Generates random stories using LLMs
Visual Consistency	Some use prompt optimization or model modification for consistency [10]	Uses AI-in-the-loop to check if image matches caption
Output Format	Mostly text + images [6,9]	Text + images + AI-generated voice narration

**Table 2:** Gesture recognition performance metrics for wave detection using smartphone gyroscope.

Metric	Value	Description	Formula	Interpretation
True Positives (TP)	28	Wave gestures correctly detected	-	28/35 intentional waves
False Negatives (FN)	7	Wave gestures missed	-	Missed detection rate
True Negatives (TN)	12	Non-gestures correctly rejected	-	12/15 natural movements
False Positives (FP)	3	Non-gestures falsely detected	-	False alarm rate
Accuracy	80.0%	Overall correct detection rate	$\frac{TP\;+\;TN}{Total}$	Balanced performance
Precision	90.3%	Detection reliability	$\frac{TP}{TP\;+\;FP}$	Low false alarms
Recall	80.0%	Wave detection sensitivity	$\frac{TP}{TP\;+\;FN}$	Moderate sensitivity
F1-Score	0.849	Harmonic mean	$2\times \frac{Precision\;\times \;Recall}{Precision\;+\;Recall}$	Overall metric
Total Trials	50	Evaluation dataset size	35 waves + 15 non-waves	Controlled testing

**Table 3:** Quantitative evaluation of semantic relevance and inter-panel visual consistency.

Sample ID	Metric Value	Category
**LLM-based semantic relevance (1–5)**		
1	3	Moderate
2	4	High
3	4	High
4	5	High
5	5	High
6	5	High
7	4	High
8	4	High
9	5	High
10	3	Moderate
**Mean**	**4.20**	–
**Std**	**0.79**	–
**CLIP-based inter-panel visual consistency**		
1	0.783	High
2	0.651	Moderate
3	0.685	Moderate
4	0.680	Moderate
5	0.787	High
6	0.726	Moderate
7	0.696	Moderate
8	0.590	Low
9	0.715	Moderate
10	0.874	High
**Mean**	**0.719**	–
**Std**	**0.080**	–

**Table 4:** End-to-end runtime stability across repeated experiments.

Metric	Value (Seconds)
Minimum time	46.46
Maximum time	61.47
Mean total time	53.98
RMSE	5.02
Number of runs	10

**Table 5:** Stage-wise end-to-end latency of the proposed system averaged across experimental runs.

Pipeline Stage	Average Time (s)	Remarks
Story generation (LLM)	∼7.0	News-to-story conversion
Image generation (four panels)	∼25.0	Diffusion-based manga panels
AI-in-the-loop refinement	∼8.0	Vision–language consistency checks
Vision model response	∼4.0	Caption/context verification
Text-to-speech synthesis	∼6.0	Audio generation and initialization
**Total end-to-end latency**	∼50–55	Gesture- to output-ready

**Table 6:** The comparison of performance metrics across different vision language models to improve the prompt and maintain consistency among all panels (qualitative ratings use a three-level scale: high/moderate/low).

Model	Time (s)	Detail Quality (Qual.)	Image Accuracy (Qual.)	Cross-Panel Consistency (Qual.)
Qwen3-VL (latest)	129.1	High	High	High
LLaMA 3.2-Vision (latest)	26.3	High	Low	Moderate
Gemma 3-Vision (latest)	21.6	High	High	High
LLaVA (latest)	8.7	Moderate	Moderate	High

**Table 7:** Qualitative evaluation of story generation quality from news inputs, including narrative consistency, semantic accuracy, and narrative detail (qualitative ratings use a three-level scale: high/moderate/low).

Model	Time (s)	Narrative Detail (Qual.)	Semantic Accuracy (Qual.)	Narrative Consistency (Qual.)
Gemma3:12b	6.76	High	High	High
LLaMA3.2:latest	6.09	High	Low	Moderate
Deepseek-r1:latest	16.41	Moderate	Moderate	Low
Mistral:latest	6.94	High	High	High
Gemma3:4b	6.43	Moderate	Moderate	Moderate
Qwen3:latest	9.0	High	High	High

## Data Availability

The datasets generated and analyzed during this study are not publicly available as they are part of an ongoing study and are currently being used for follow-up experiments. Requests to access the datasets should be directed to the corresponding author.
